# Online Work Force Analyzes Social Media to Identify Consequences of an Unplanned School Closure – Using Technology to Prepare for the Next Pandemic

**DOI:** 10.1371/journal.pone.0163207

**Published:** 2016-09-21

**Authors:** Jeanette J. Rainey, Jasmine Kenney, Ben Wilburn, Ami Putman, Yenlik Zheteyeva, Megan O’Sullivan

**Affiliations:** 1 Division of Global Migration and Quarantine, Centers for Disease Control and Prevention, Atlanta, GA, United States of America; 2 Oak Ridge Institute for Science and Education (ORISE), Oak Ridge, TN, United States of America; Pfizer Inc, UNITED STATES

## Abstract

**Background:**

During an influenza pandemic, the United States Centers for Disease Control and Prevention (CDC) may recommend school closures. These closures could have unintended consequences for students and their families. Publicly available social media could be analyzed to identify the consequences of an unplanned school closure.

**Methods:**

As a proxy for an unplanned, pandemic-related school closure, we used the district-wide school closure due to the September 10–18, 2012 teachers’ strike in Chicago, Illinois. We captured social media posts about the school closure using the Radian6 social media-monitoring platform. An online workforce from Amazon Mechanical Turk categorized each post into one of two groups. The first group included relevant posts that described the impact of the closure on students and their families. The second group included irrelevant posts that described the political aspects of the strike or topics unrelated to the school closure. All relevant posts were further categorized as expressing a positive, negative, or neutral sentiment. We analyzed patterns of relevant posts and sentiment over time and compared our findings to household surveys conducted after other unplanned school closures.

**Results:**

We captured 4,546 social media posts about the district-wide school closure using our search criteria. Of these, 930 (20%) were categorized as relevant by the online workforce. Of the relevant posts, 619 (67%) expressed a negative sentiment, 51 (5%) expressed a positive sentiment, and 260 (28%) were neutral. The number of relevant posts, and especially those with a negative sentiment, peaked on day 1 of the strike. Negative sentiment expressed concerns about childcare, missed school lunches, and the lack of class time for students. This was consistent with findings from previously conducted household surveys.

**Conclusion:**

Social media are publicly available and can readily provide information on the impact of an unplanned school closure on students and their families. Using social media to assess the impact of an unplanned school closure due to a public health event would be informative. An online workforce can effectively assist with the review process.

## Introduction

Closing schools can help slow influenza transmission among a school-aged population and is an important mitigation measure during the early stages of an influenza pandemic [1–4]. However, unplanned school closures can also cause economic and social costs and consequences for students and their families [1], especially if the closure lasts for several weeks or more. Public health officials must therefore carefully evaluate the balance between the benefits and the consequences of these closures to mitigate the spread pandemic influenza [1, 5]. Being aware of these costs and consequences during the course of a pandemic could assist public officials to better evaluate this balance.

Since influenza pandemics are infrequent in the United States, public health officials have relied on investigating the costs and consequences of unplanned school closures due to non-pandemic related causes. Although many of these investigations have been informative, some were implemented several months after schools reopened [6–8], possibly resulting in recall bias. Others were conducted during infectious disease outbreaks or immediately after, but the investigations still required time and resources for planning, implementation, and analysis before results became available [9–11]. During the 2009 Influenza A (H1N1) pandemic, telephone polls were conducted to identify challenges to families associated with recent school closures. These polls were substantially more time-efficient but only captured information for a single point in time [12]. All types of household surveys and telephone polls create some level of burden on household respondents.

The accessibility and popularity of social media such as Twitter, Facebook, and blogs provide a new opportunity to assess public perception and the impact of community-wide events [13–21]. In a 2014 study, 74% of online adults used social media [22], suggesting that a high volume of potentially relevant information is available in the public domain. These sources could capture nearly real-time information (in relation to the community event) in unconstrained formats while minimizing common biases (e.g., recall bias) and the burden associated with traditional survey methods [13–18, 23–24]. During a pandemic, monitoring public perception and sentiment over time could help determine when modifications or different strategies may be needed for mitigating disease transmission.

We analyzed social media related to the district-wide unplanned school closure due to the September 2012 Chicago teacher’s strike, which affected more than 400,000 students attending the 600 elementary, middle, and high schools in the district [25]. Our objectives included: 1) evaluating whether social media could be used to identify costs and consequences as effectively as traditional household surveys and telephone polls; 2) determining if the costs and consequences identified varied over the duration of an unplanned school closure; and 3) exploring the use of an online workforce as a way to efficiently review and interpret relevant social media posts. This type of workforce could be easily leveraged if needed during a future pandemic or other public health emergency.

## Methods

### Data collection

We used the Radian6 (San Francisco, CA) social media-monitoring platform to retrospectively capture social media posts about the Chicago City School District closure occurring from September 10–18, 2016. Since the strike was anticipated prior to the first day of the closure, we captured social media posts from Twitter, Facebook, blogs, forums, and comments between September 8 and September 21, (two days before the strike started to three days after the strike ended). We used the following combination of exact search terms: “strike Chicago” AND “breakfast” OR “childcare” OR “daycare” OR “lunch” OR “parent”. A proximity score of “5” was applied to the terms “strike” and “Chicago” (on a scale of 1–20, with 1 being exact [i.e., strike and Chicago together]). We included childcare and missed free or reduced-priced school lunches since these issues were identified in previous unplanned school closure investigations. Several different combinations of search terms were tested to capture relevant information while limiting unnecessary noise (e.g., posts related to political aspects of the strike). With the above Boolean logic, only “strike Chicago” and one of the other terms were required in any of the identified posts.

We downloaded the content, platform, date, and time of all social media posts meeting the search criteria. Social media not written in English, in non-ASCII script, or sent by a client identified as an application program interface (API) (usually automatically generated and therefore considered to be “spam”) were excluded. We included re-postings of social media since they reflect the sharing of similar information and sentiment. Our unit of analysis was a single post (or re-post) from the types of social media included in the initial search. Radian6 captures social media-specific sites by using a combination of RSS feeds, proprietary crawlers, and API access for certain sources such as Twitter and Facebook. Radian6 adheres to the terms of use for each social media source (http://www.exacttarget.com/blog/dear-radian6-howd-you-get-that-data/).

Social media posts were categorized as “relevant” (related to how the school closure affected students or their families) or “irrelevant” (related to political aspects of the strike, status of the education and welfare system in Chicago, or an unrelated event in Chicago or elsewhere). Each post was reviewed and categorized by five different workers recruited through Amazon Mechanical Turk, an online marketplace of workers to perform various Human Intelligence Tasks (https://www.mturk.com/mturk/welcome). Within Mechanical Turk, we developed an online customized categorization project that included instructions, definitions, and examples using a few lines of JavaScript. For data-management purposes, we uploaded social media posts in batches of 200 each and only included the post content and unique identification number (username, date, and social media source were excluded). We limited eligible Turk workers to those residing in the United States with a master rating (scoring >99% on previous categorization tasks). Workers were able to review as many or as few posts as possible but could review each individual post only once, as verified through a worker’s unique identification number. Individual posts could therefore be reviewed and categorized by different sets of five Turk workers. The categorization of each post as relevant or irrelevant was based on agreement among four of five workers (≥ 80%).

Posts with poor agreement among the Turk workers (< 80%) were subsequently reviewed by four subject matter experts (SMEs) comprising public health staff with training in reviewing social media posts and investigating unplanned school closures. The final categorization of these posts was determined by a majority of these experts. In the SME review, we included a 5% random sample of posts (n = 180) previously categorized as irrelevant by the Turk workers to assess the agreement between the SMEs and recruited Turk workers. All posts categorized as relevant by Turk workers were also reviewed and verified by the SMEs.

### Sentiment analysis

Using a combination of context (e.g., school lunches) and semantics (e.g., wonderful/terrible), each relevant post was categorized into one of three sentiment groups [16, 19]: positive, negative, or neutral. These groups were defined as follows:

1. Positive: The author expressed a good or favorable experience as a result of the closure. Example of positive post: “Another day without school, a day to play.”
2. Negative: The author expressed inconveniences or an undesirable effect as a result of the closure. Example of negative post: “I can’t find childcare.”
3. Neutral: The author did not express any particular sentiment. Example of neutral post: “Schools will be open at 8:00 to serve breakfast to students.”

We tested and modified project definitions before initiating the sentiment analysis to maximize clarity and understanding. Posts stating “school will be closed today” or describing the availability of services were considered statements of fact and were categorized as neutral. When both positive and negative sentiments were expressed, the post was categorized as negative since our overall objective was to describe the costs and consequences of an unplanned school closure.

We described the temporal trends of relevant posts and their sentiments to assess whether perceptions and sentiments changed during the 10-day closure. We also abstracted up to three consequences from each post categorized as having a negative sentiment. We qualitatively compared these findings with results from recent household surveys and a telephone poll following other unplanned school closures. The similarities and differences were used to assess the validity of our approach. Additionally, we calculated overall and daily sentiment scores as the difference between the number of positive and negative sentiment posts divided by the sum of all relevant posts ((positive—negative)/(positive + negative + neutral)) [16]. A score less than zero suggested negative sentiment, while a score greater than zero suggested positive sentiment [16]. Data were analyzed using SAS (version 9.3, Cary, NC).

When an author’s privacy settings are turned off, opinions expressed through social media are considered public information. Certain forms of social media are tagged with personal identifiers (e.g., profile name on Twitter and Facebook), which are publicly available but were not included in the analysis. Access to the information captured for this project using Radian6 adhered to the terms of use for each source of social media. The project protocol was reviewed and approved by the Centers for Disease Control and Prevention’s Human Subjects Research Office (HSRO). Since we relied on publicly available data and there was no contact with social media users, the HSRO determined that the project was exempt from review by the Institutional Review Board.

## Results

We retrospectively captured 4,546 social media posts for the dates of September 8, 2012 to September 21, 2012 using our search criteria. Of these, 930 (20%) were categorized as relevant in describing the impact of the strike-related closure on students and their families (Table 1). The remaining 3,616 (80%) social media posts were excluded as irrelevant. The 4,546 posts were reviewed by 301 different Turk workers (each categorizing 1 to 1,532 posts such that every post was reviewed by five different workers). The median number of posts categorized per worker was 17 (IQR: 3 to 71), and the median time for categorizing each post was 1.7 minutes (IQR: 13 seconds to 5 minutes). On average, Turk workers completed the review of each batch of 200 posts within 2–3 hours. The agreement between Turk workers and SMEs was 99% for irrelevant posts (two of the subset of 180 posts were re-categorized as relevant by experts) and 98% for relevant posts (16 of 944 posts were re-categorized as irrelevant). Almost 90% of the 930 relevant posts were from Twitter, blogs and comments, Facebook discussions, and mainstream media comments (Table 1).

**Table 1:** Distribution of relevant and irrelevant posts by social media type. Relevant posts further categorized according negative, positive, and neutral sentiment. Posts captured from social media referencing Chicago teachers’ strike from September 8–21, 2012 (two days before to three days after strike).

Social Media Type	Irrelevant (%)	Relevant (%)	Negative (%)	Positive (%)	Neutral (%)
**Twitter**	273 (7)	266 (29)	138 (22)	8 (16)	120 (46)
**Blogs and Comments**	938 (26)	232 (24)	158 (25)	8 (16)	66 (25)
**Facebook Discussions**	528 (15)	165 (18)	117 (19)	5 (10)	43 (17)
**Mainstream Media Comments**	1068 (30)	147 (16)	116 (19)	17 (33)	14 (5)
**Forum Posts and Replies**	736 (20)	114 (12)	84 (14)	13 (25)	17 (7)
**YouTube and Comments**	47 (1)	2 (<1)	2 (<1)	0 (0)	0 (0)
**Other**^a^	26 (1)	4 (<1)	4 (<1)	0 (0)	0 (0)
**Total**	3,616	930	619	51	260

^a^Other includes Google, LiveJournal, WSJ, fc2.com, multiply.com, tumblr.com, typepad.com, livedoor.jp.blogs, and Sina Blog.

Of the relevant posts, 619 (67%) were further categorized as expressing negative sentiment, 51 (5%) were positive, and 260 (28%) were neutral. We abstracted 1,007 costs and consequences from the 619 negative posts. The most frequently expressed negative sentiment involved concerns about finding childcare or the cost of childcare (n = 377), missing school or class time (n = 210), and missing free or reduced-priced school meals (n = 185) (Table 2). One parent posted, “both of us are working, I have no relatives, nobody I can turn to in town,” while another expressed, “besides the daycare issue, they just need to be in school.” These costs and consequences were consistent with findings from other unplanned school closure investigations (Table 3). Additional consequences were captured for the unplanned school closure in Chicago, including concerns with child safety and city violence (n = 45). Social media provided information for parents on locating childcare and alternative meal services for students (e.g., “CTU has announced their strike. Visit http://t.co/TVptuuTm or call 311 if you have no alternative childcare tomorrow.”).

**Table 2:** Costs and consequences abstracted from 619 social media posts expressing negative sentiment. Each post could express up to three costs and consequences. Posts captured from social media referencing Chicago teachers’ strike from September 8–21, 2012 (two days before to three days after strike).

Primary consequence and sub-category	Frequency
**General**	
** Disrupted routines (e.g., scrambling, in turmoil)**	123
** Stressed/frustrated parents**	33
** General burden**	26
** Uncertainty about length of closure**	8
***Total***	*190*
**Childcare**	
** Problems finding childcare/places for children to go**	164
** General childcare concerns—single/working parents**	108
** Cost of childcare**	50
** Loss of work time/pay to stay home with children**	35
** Concern for job, benefits, or other work issues**	15
** Adjusting work schedule due to childcare**	5
***Total***	*377*
**Missed School**	
** No classwork/learning**	99
** Students should be in school**	56
** Hard on students (disruption of school year)**	35
** Rights to an education**	20
***Total***	*210*
**School services**	
** Missing free/reduced priced meals**	185
***Total***	*185*
**Student Safety**	
** Student public health, safety, and welfare**	17
** Students in streets**	11
** Lack of supervision**	8
** City/gun violence and crime**	7
** Gang activity**	2
***Total***	*45*

**Table 3:** Qualitative comparison of the Chicago teacher’s strike social media findings with results from traditional household surveys in Mississippi, Colorado, and Kentucky and a telephone poll about the costs and consequences of unplanned school closures^a^.

Information captured	Chicago, Illinois, 2012	Harrison County School District, Mississippi, 2012^d^	Suburban Denver School District, Colorado, 2013^e^	Rural School District, Kentucky, 2013^f^	Harvard Poll, June 2009^g^
**Cause of unplanned school closure**	Teachers’ Strike	Hurricane Preparation	Absenteeism-influenza-like illness	Absenteeism-influenza-like illness	Pandemic Influenza
**Duration (school days)**	7	4	5	4	1–5^h^
**Consequences identified**					
** Problems finding child care**	YES	YES	YES	YES	YES
** Missed work/pay**	YES	YES	YES	YES	YES
** Missed free/reduced priced school lunches**	YES	YES	YES	YES	YES
** Missed class time**	YES	YES	NO	NO	NO
** Student safety/gangs**	YES	NO	NO	NO	NO
**Uncertainty about length of closure**	YES	YES	YES	YES	YES
**Representativeness**^b^	NO	YES	YES	YES	YES
**Community response**^c^	YES	NO	NO	NO	NO
**Sentiment available in real-time**	YES	NO	NO	NO	NO

^a^Includes primary consequences only.

^b^Data available to estimate the percentage of the target population experiencing the same or similar costs and consequences.

^c^Use of social media for communicating availability of services for families impacted by the strike (e.g., school district shared information about where to find alternative childcare).

^d^Unpublished report from Mississippi unplanned school closure investigation, Centers for Disease Control and Prevention, 2014.

^e^Epson EE, Zheteyeva YA, Rainey JJ, Gao H, Shi J, Uzicanin A, Miller L (2015) Evaluation of an Unplanned School Closure in a Colorado District: Implications for Pandemic Influenza Preparedness. *Disaster Med Public Health Prep* 9:4–8.

^f^Russell ES, Zheteyeva YA, Gao H, Shi J, Rainey JJ, Thoroughman D, Uzicanin A (2016) Reactive School Closure during Increased Influenza-like-Illness (ILI) Activity in Western Kentucky, 2013; A Field Evaluation of Effect on ILI Incidence and Economic Social Consequences for Families. Open Forum Infectious Diseases 3(3):doi: 10.1093/ofid/ofw113.

^g^Centers for Disease Control and Prevention (2010) Parental attitudes and experiences during school dismissals related to 2009 Influenza A (H1N1)—United States, 2009. *MMWR Morb Mortal Wkl* 59:1131–4.

^h^Approximately 9% of schools were closed for >5 days.

**Fig 1:**
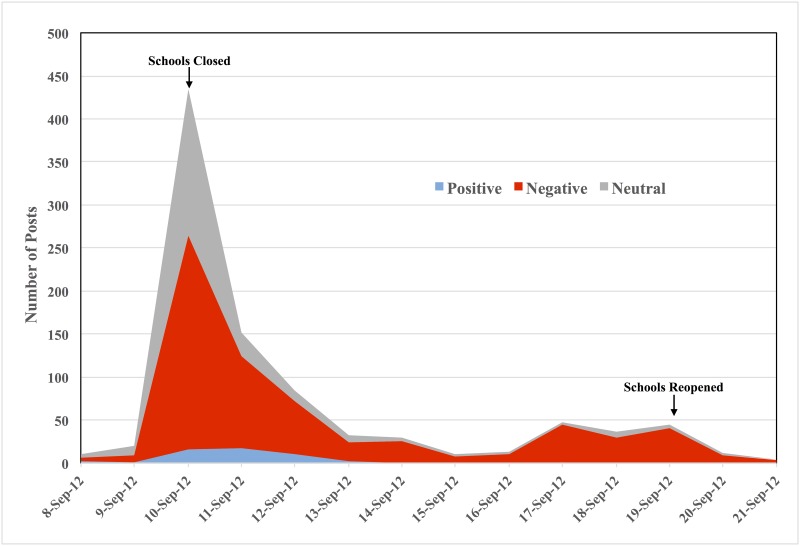
Number of relevant posts identified from social media by sentiment^a^ and date. Relevant posts mentioned impact of unplanned school closure due to Chicago teachers’ strike from September 8–21, 2012 (two days before and three days after strike) on students and their families (n = 930). ^a^Sentiment definitions: Positive: The author expressed a good or favorable experience as a result of the closure. Example of positive post: “Another day without school, a day to play.” Negative: The author expressed inconveniences or undesirable effect as a result of the closure. Example of negative post: “I can’t find childcare”. Neutral: The author did not express any particular sentiment. Example of neutral post: “Schools will be open at 8:00 to serve breakfast to students”.

**Fig 2:**
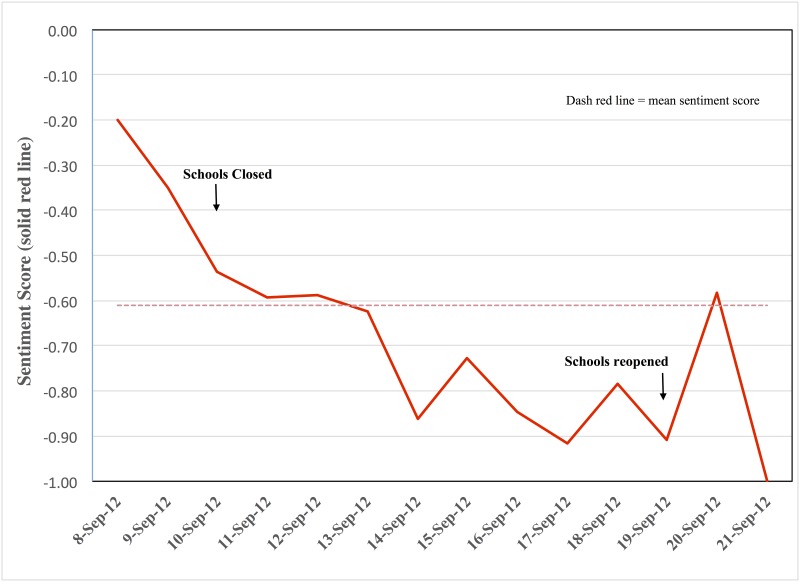
Sentiment score of relevant posts identified from social media by date. Relevant posts mentioned impact of unplanned school closure due to Chicago teachers’ strike from September 8–21 (two days before to three days after strike) on students and their families (N = 930). Sentiment score was calculated as: (positive posts—negative posts)/(positive posts + negative posts + neutral posts)^a-b^. ^a^Sentiment score < 0 suggests negative sentiment; score > 0 suggests positive sentiment. ^b^Score on September 21 reflects only four relevant posts, all expressing negative sentiment.

The number of all relevant posts and those expressing negative sentiment was greatest on day 1 of the strike (September 10, 2012) and decreased dramatically by day 3 (September 13, 2012 [Fig 1]). The sentiment score was estimated to assess the relative change in negative versus positive sentiment over time. During the Chicago teachers’ strike, the mean sentiment score was -0.61 (daily range: -0.20 to -1.0 [Fig 2]).

## Discussion

Reviewing and analyzing social media posts at the time of the Chicago teachers’ strike generated information on the consequences and community sentiment of this unplanned district-wide school closure. The types of identified consequences were similar to findings from previous unplanned school closure investigations and included missing work, childcare costs, and lack of access to free and reduced-priced school lunches [6–9]. We also captured social media voicing concerns about child safety and street violence, neither of which had been previously identified as possible consequences of unplanned school closures. These newly identified concerns and consequences may highlight the importance of capturing local information when assessing the impact of unplanned school closures on students and their families. The unstructured format of social media posts can elicit information not previously anticipated [13–17, 23], and is likely an added benefit of social media when compared to traditional surveys and telephone polls.

We were also able to capture information on the costs and consequences as well as sentiment in near real time throughout the strike. Relevant posts and negative posts were most frequent during the first day of the strike and decreased dramatically by day 3 (September 12), suggesting that concerns and challenges can vary over the duration of an unplanned school closure. This variability could reflect that parents were initially unprepared for the closure but were able to adapt rapidly to the unanticipated disruption (including help from information shared on social media about access to services). The decrease could also indicate that the public quickly experienced information saturation with a subsequent decrease in interest [26]. Similar trends in social media were identified before, during, and after the March 2011 Japanese earthquake and tsunami [27]. The lack of a perceived direct benefit from the strike may have partially contributed to the consistently negative sentiment score (mean score was -0.61). In general, households are more likely to support unplanned school closures when there is a perceived benefit to the health and wellbeing of the student, regardless of whether the closure is implemented pre-emptively (prior to widespread disease transmission) or reactively (after a larger percentage of students and teachers are already ill) to an infectious disease outbreak [8, 28–30]. We also hypothesize that negative sentiment is more likely to be expressed on social media than positive or neutral sentiment. Social media could serve as an outlet for expressing frustration by a certain but small segment of the population. Background information on the impacted population, such as the percentage of students belonging to single parent households or eligible for free or reduced priced school meals, therefore, could be helpful in interpreting social media findings.

During an influenza pandemic, public health officials will need to rapidly capture and respond to perceived challenges in the implementation of mitigation strategies, including school closures. We explored the use of Amazon Mechanical Turk’s online workforce as a way to rapidly and accurately review and categorize social media posts. To ensure quality results, we requested “master workers” residing in the United States who had a previous rating of 99% or greater on previous projects. This improved the likelihood that Turk workers would be familiar with school closures and related challenges for families and students in the United States, as well as the possible use of American slang and language nuances. On average, five different Turk workers were able to complete the review of each batch within 2–3 hours with relatively good accuracy (>95% for both relevant and irrelevant posts). The number of unique workers participating in the review supports the scalability of this approach. Using such a workforce during a pandemic could compare favorably to household surveys that typically require weeks to months for researchers to collect, clean, and analyze available data. Although a number of machine learning tools are available for automating this process [16, 31], a combination of approaches will likely be needed due to the complexity of language (e.g., sarcasm), as well as misspellings and grammatical errors in social media [16, 19].

We selected the Chicago teachers’ strike for this initial social media project due to the length of the unplanned school closure and the size of the impacted school district (>400,000 students). Although social media use is higher among young adults and in urban and suburban areas [22], use appears to be independent of education level and race or ethnicity. Therefore, we anticipated that this closure would elicit a high volume of commentary on social media. We captured over 4,500 social media posts during the project period. Due to the nature of the closure, only 20% of the posts were relevant to assessing the impact of the closure on students and their families. Although many of these relevant posts included re-tweets and shares, we did not have access to the metrics required to estimate the specific frequency. The majority of posts were categorized as irrelevant since they addressed the political nature of the strike, the role of unions, and general concerns regarding the welfare and education systems in Chicago. More posts could be relevant or positive if captured during a public health-related closure, where community comments would likely include the perceived health benefits in addition to the costs and consequences of the closure.

We attempted to increase the percentage of posts meeting our definition of “relevant” by including the terms “breakfast,” “childcare,” “daycare,” “lunch,” and “parent” in the Radian6 search criteria. We primarily included these terms to minimize the amount of “noise” related to the political nature of the closure [13–15]. We propose that the any bias resulting from the inclusion of these terms was limited since only one of the terms was required using Boolean logic. Our findings related to child safety and street violence (which were not search terms) support the use of this approach. We also used exact matching for “breakfast,” “childcare,” “daycare,” “lunch,” or “parent” and proximity for “Strike” and “Chicago”. Relevant posts including abbreviations and misspellings could have been missed (e.g., day care). In a qualitative review of relevant posts, however, a large percentage of childcare-related posts referenced childcare as two words. A number of other challenges were identified in categorizing social media posts, including complex posts referencing both political aspects of the strike and the availability of non-educational services to students during the school closure. Additionally, the combination of context and semantics to assign sentiment can involve a level of human subjectivity. To minimize these challenges, we pilot-tested our categorization and sentiment definitions. However, neither the categorization nor sentiment analysis was likely to be 100% accurate.

Despite increased access to social media, information from these sources is likely to be unrepresentative [13–17]. We could only partially limit the geographic scope of social media users with Radian6, and we were unable to determine whether an author of a post was a student in or had a child attending a Chicago City District-school. Our findings could have inadvertently included posts from persons not directly impacted by the Chicago school closure. Capturing IP addresses from social media users when available could help identify the author’s physical location and further assist public health officials in addressing identified negative impacts where and when they occur [13–18, 23, 26–27].

## Conclusion

Social media can provide information about the costs and consequences of an unplanned school closure on students and their families. Social media posts are publicly available and can be captured in near real-time to monitor changes in sentiment over time. Social media can also capture the costs and consequences not identified through traditional approaches. The political nature of the teachers’ strike likely influenced the topics and sentiment expressed in this project. Future projects using social media to assess the impact of a public health-related school closure and to capture information on authors’ physical location would be informative. An online workforce possibly combined with new machine learning tools could further improve the capacity to rapidly identify and interpret relevant social media posts. This approach could help public health officials more effectively monitor and balance the anticipated health benefits with possible costs and consequences of unplanned school closures during a future pandemic.
